# Evaluating Inclusion of Commercial Pistachio By-Product as a Functional Ingredient in Rainbow Trout Fishmeal and Plant Meal-Based Diets

**DOI:** 10.3390/antiox13111280

**Published:** 2024-10-23

**Authors:** Mosope F. Abanikannda, Mark B. Shiflett, Ana Rita C. Morais, Jeoungwhui Hong, Wendy M. Sealey, Jacob W. Bledsoe

**Affiliations:** 1 Department of Animal Veterinary & Food Sciences, University of Idaho, Moscow, ID 83844, USA; mosopea@uidaho.edu; 2 Aquaculture Research Institute, Hagerman Fish Culture Experiment Station, University of Idaho, Hagerman, ID 83332, USA; jhong@chonnam.ac.kr; 3 Wonderful Institute for Sustainable Engineering, University of Kansas, Lawrence, KS 66045, USA; mark.b.shiflett@ku.edu (M.B.S.); ana.morais@ku.edu (A.R.C.M.); 4 US Department of Agriculture (USDA) Agriculture Research Service (ARS), Bozeman, MT 59715, USA; wendy.sealey@usda.gov

**Keywords:** sustainable aquaculture, antioxidants, functional feed ingredients, pistachio shell powder, gut microbiome, rainbow trout

## Abstract

To meet the growing demand for sustainable aquaculture, plant proteins are being explored as alternative sources in fish diets. However, some plant proteins can have adverse health effects on fish, prompting research into functional feed ingredients to mitigate these issues. This study investigated pistachio shell powder (PSP), rich in antioxidants, as a functional feed ingredient for rainbow trout (*Oncorhynchus mykiss*). The effects of PSP inclusion (0%, 0.5%, 1%, 2%) on growth performance, intestinal health, and gut microbiota were assessed in fish fed either a fishmeal (FM) or plant meal (PM) diet over a 12-week feeding period. The results indicated that PSP inclusion at 1% significantly (*p* < 0.05) improved weight gain and growth performance in FM treatments, with no impact on growth in PM treatments. No significant differences were observed in other growth parameters, intestinal morphology, or oxidative stress markers, although a trend toward the downregulation of inflammatory genes was noted in PM treatments at 2% PSP inclusion. PSP inclusion did not significantly alter gut microbiota alpha diversity but affected beta diversity at the 0.5% level in the FM treatments (*p* < 0.05). Differential abundance analysis of gut microbiota revealed taxa-specific responses to PSP, particularly the genus *Candidatus arthromitus*, increasing in relative abundance with PSP inclusion in both the FM- and PM-based treatments. Overall, PSP inclusion up to 2% did not have significant adverse effects on the growth, intestinal health, or antioxidant status of rainbow trout.

## 1. Introduction

Aquaculture is a rapidly growing sector within the global food industry, offering a sustainable solution to meeting the increasing demand for animal proteins. Recently, the contribution of salmonids to global aquaculture production has seen a marked increase. Specifically, rainbow trout (*Oncorhynchus mykiss*) accounts for approximately 1.5% of the total global finfish production in inland aquaculture systems, while Atlantic salmon constitutes about 32.6% of finfish production within marine and coastal aquaculture settings [1]. Amidst the rising production of salmonids in aquaculture, the production of these carnivorous fish is faced with significant challenges due to the relatively high costs of conventional feed ingredients, particularly fishmeal and fish oil. To address these economic constraints, nutritionists have been exploring plant-based diets as a means to reduce feed expenses. However, plant-based diets have been linked to several adverse effects in carnivorous fish like rainbow trout, including enteritis triggered by soybean products [2,3,4] and an increased production of reactive oxygen species (ROS) resulting from plant-based oils [5]. In response to these challenges, researchers have embarked on developing different solutions. These include the selective breeding of rainbow trout strains with higher tolerance to plant-based diets [6,7], as well as the incorporation of specific enzymes and additives into the feeds to improve the digestion and assimilation of complex plant-derived nutrients [8,9]. Despite their potential, these approaches may require considerable time and financial investment. A more widely accepted approach involves incorporating dietary supplements into fish diets [10,11,12].

Over the past few years, pistachio production in the U.S. has increased significantly, with the U.S. contributing around 51% (334,152 tons) of global pistachio production in 2020 [13]. Of the pistachios sold for human consumption in the U.S., roughly 50% of the mass from the harvest is considered by-product. Without alternative uses, these by-products would be disposed of as waste [14]. In 2020, more than 82% of U.S. pistachio production, amounting to approximately 865 million pounds, was marketed as shelled products [15]. Pistachio shell waste is a by-product of processing pistachio nuts, which are rich in biologically active compounds like antioxidant polyphenols present in the shells and skins [14,16] and prebiotic polysaccharides found in both the hulls and shells [17,18]. Such components suggest that pistachio by-products could serve as functional dietary supplements in aquafeeds, potentially offering a cost-effective and sustainable alternative to conventional feed ingredients while mitigating the adverse effects associated with plant-based diets in carnivorous aquaculture species. Although little is known about the proximate composition of pistachio shells and their use in animal nutrition, research into other organisms on the use of pistachio nuts has highlighted their potential to have beneficial effects on gut health [16,19,20]. Recent studies report a fiber content of around 10% of a pistachio nut’s dry weight [21]. This high fiber content contributes to various health benefits in humans, including improved digestion and the modulation of the gut microbiota [21]. Additionally, the high content of antioxidants in pistachios may mitigate the negative effects of reactive oxygen species (ROS) in the gut. These antioxidants can reduce ROS production, thereby preventing the formation of by-products that act as terminal electron acceptors, which could promote the overgrowth of certain facultative anaerobes and lead to gut dysbiosis [22,23,24]. Concurrently, their high fiber content may enhance microbial stability within the gut, promoting the proliferation of beneficial commensal bacteria [21,25,26,27]. This dual action of antioxidants and fiber could potentially contribute to maintaining a balanced and healthy gut microbiome by reducing oxidative stress and promoting the growth of beneficial bacteria. However, there are currently limited studies evaluating the efficacy of dietary supplementation with pistachio by-products in omnivorous species [28,29,30], and no studies have been conducted on carnivorous fish species.

The objective of this study was to determine the dose-dependent effects of pistachio shell powder (PSP) supplementation on the growth performance, oxidative and inflammatory gene expression, and gut health of rainbow trout fed either a traditional fishmeal (FM) diet or an ultramodern plant meal (PM)-based diet.

## 2. Materials and Methods

### 2.1. Experimental Diets and Fish Husbandry

A total of eight diets were prepared and extruded into 4.5 mm pellets at the Bozeman Fish Technology Center, MT, USA. Diets within each protein group were formulated to be isonitrogenous, isolipidic, and isocaloric (Table 1), with four diets in each protein category (fishmeal vs. plant meal). The base control diets for each protein category (FM1 and PM1) had 0% PSP inclusion. The subsequent diets contained increasing levels of PSP: FM2 and PM2 had 0.5% PSP, FM3 and PM3 had 1% PSP, and FM4 and PM4 had 2% PSP inclusion. The formulation of these experimental diets was guided by established protocols from the existing literature on the optimal nutrient requirements for rainbow trout [31,32,33,34].

A twelve-week feeding trial was conducted at the University of Idaho Aquaculture Research Institute, Hagerman, USA, following approval from the University of Idaho’s Institutional Animal Care and Use Committee (IACUC-2022-41).

A total of 840 rainbow trout fingerlings (initial average live weight of 19.15 ± 0.25 g/fish) were sourced from a commercial germplasm line. Thirty-five fish were randomly stocked in one of twenty-four 1300 L tanks. A flow-through system with a spring water source was maintained at a constant temperature of 15 °C and a flow rate of 8–10 L min^−1^. pH, nitrite, and ammonia were periodically monitored using a LaMotte kit (LaMotte company, Chestertown, MD, USA) and remained well within the acceptable range. Dissolved oxygen was measured using a YSI Pro 20 DO meter (Xylem Inc, Yellow Springs, OH, USA) and was maintained at >6.5 mg/L.

During the trial, fish were hand-fed to apparent satiation three times daily following established recommendations for optimal feeding frequency in trout production [35], and feed consumption was recorded by tank. Rearing tanks were maintained under strict hygiene protocols, with bi-weekly cleanings, while waste products were removed via flow-through exchange. Every three weeks, bulk fish weights were recorded in each tank to track growth.

Growth and feed consumption data were used to calculate average weight gain, feed conversion ratio, specific growth rate, daily growth index, protein efficiency ratio, protein retention, energy retention, and survival using the formulas described by Lugert et al. [36] and Hong et al. [37], as shown below.

$$Feed\;Conversion\;Ratio\;\left(FCR\right)=\frac{Feed\;\mathrm{Consumption}\;(g)}{(\mathrm{final}-\mathrm{initial}\;\mathrm{biomass}\;(g)+\mathrm{dead}\;\mathrm{fish}\;\mathrm{weight}\;(g))}\times 100$$

$$Specific\;Growth\;Rate\;\left(SGR\right)=\frac{\ln\left(\mathrm{Final}\;\mathrm{Weight}\right)-\ln\left(\mathrm{Initial}\;\mathrm{Weight}\right)}{\mathrm{Time}\;(\mathrm{days})}$$

$$Daily\;Growth\;Index\;\left(DGI\right)=\frac{\left({\mathrm{Final}\;\mathrm{Weight}}^{\frac{1}{3}}-{\mathrm{Initial}\;\mathrm{Weight}}^{\frac{1}{3}}\right)}{\mathrm{Time}\;(\mathrm{days})}\times 100$$

$$Average\;Weight\;Gain=\frac{\mathrm{Final}\;\mathrm{Weight}-\mathrm{Initial}\;\mathrm{Weight}}{\mathrm{Number}\;\mathrm{of}\;\mathrm{Fish}}$$

$$Protein\;Efficiency\;Ratio\left(PER\right)=\frac{\mathrm{Weight}\;\mathrm{Gain}\;(g)}{\mathrm{Protein}\;\mathrm{Intake}\;(g)}$$

$$Protein\;Retention=\left(\frac{\mathrm{Protein}\;\mathrm{in}\;\mathrm{Final}\;\mathrm{Body}\;\mathrm{Composition}\;(g)-\mathrm{Protein}\;\mathrm{in}\;\mathrm{Initial}\;\mathrm{Body}\;\mathrm{Composition}\;(g)}{\mathrm{Protein}\;\mathrm{Intake}\;(g)}\right)\times 100$$

$$Energy\;Retention=\left(\frac{\mathrm{Energy}\;\mathrm{in}\;\mathrm{Final}\;\mathrm{Body}\;(\mathrm{cal})-\mathrm{Energy}\;\mathrm{in}\;\mathrm{Initial}\;\mathrm{Body}\;(\mathrm{cal})}{\mathrm{Energy}\;\mathrm{Intake}\;(\mathrm{cal})}\right)\times 100$$

$$Survival\;Rate\left(\%\right)=\left(\frac{\mathrm{Number}\;\mathrm{of}\;\mathrm{fish}\;\mathrm{at}\;\mathrm{the}\;\mathrm{end}\;\mathrm{of}\;\mathrm{trial}}{\mathrm{Initial}\;\mathrm{Number}\;\mathrm{of}\;\mathrm{fish}}\right)\times 100$$

### 2.2. Sample Collection

At the conclusion of the twelve-week study, three fish from each tank were sampled for the collection of biological samples following euthanasia via overdose with MS-222. Sample collection included (1) mucosa from the distal intestine for microbiome analysis, (2) distal intestinal tissue for gene expression analysis and histology, and (3) whole blood collected by caudal venipuncture to evaluate the physiological effects of PSP inclusion in serum. In addition, three fish from each tank were sacrificed for whole-body proximate analysis to evaluate the effects of PSP on gross nutrient assimilation and retention.

### 2.3. Isolation of Serum for TAC and TPC Assays

At the end of the study, 2 mL of whole blood was collected from three fish per tank (a total of 72 blood samples) using syringes. Blood samples were centrifuged at 1500× *g* for 10 min at 4 °C. The resulting supernatant was transferred into a new 1.5 mL tube and stored at −80 °C for TAC and TPC analyses.

### 2.4. Diet Extraction for TAC and TPC Assays

For the TAC assay, diet and PSP samples were processed according to the method outlined by Kiron et al. [38]. The samples were homogenized in deionized water at a ratio of 1:2 (*w*/*v*) and centrifuged at 10,000× *g* for 10 min at 4 °C. The resulting supernatant (water-soluble fraction) was recovered and stored. The remaining insoluble fraction (pulp) was further extracted with pure acetone at a ratio of 1:4 (*w*/*v*), mixed at room temperature for 60 min, and centrifuged again at 10,000× *g* for 10 min at 4 °C. The combined results from the water-soluble fraction and acetone extract were used to determine TAC values.

For the TPC assay, diet and PSP samples were processed according to the manufacturer’s protocol (Abcam: ab273293 Phenolic Compounds Assay Colorimetric Kit) by homogenizing in a methanol/H_2_O/1NHCl solution (70:29.5:0.5) at 37 °C for 4 h. The mixture was then briefly centrifuged at 3000× *g* for 30 s at 4 °C. The resulting supernatant was diluted to a 2x concentration and then processed for TPC analysis.

### 2.5. TPC Assay

The total phenolic compound (TPC) content was measured using a TECAN Infinite M200 PRO (Tecan US Inc, Morrisville, NC, USA) instrument with half-area, clear, 96-well flat-bottom plates (Corning Incorporated, ME). Duplicate aliquots of 25 µL of raw serum from each of the 72 samples, as well as 25 µL of the homogenized diet and PSP samples, were processed in duplicate using the ab273293 Phenolic Compounds Assay Colorimetric Kit (Abcam, Cambridge, MA, USA) according to the manufacturer’s protocol. After the assay was run, absorbance readings were collected, and the catechin equivalents, according to the standards, were collected. A catechin standard curve was generated from each assay run, and the regression equation was used to determine the catechin equivalents for each sample. An R^2^ value greater than 0.98 was maintained for all assays. The total concentration of TPC for each sample was calculated as ((B × D)/V) = nmol/µL, where

$$\mathrm{Total}\;\mathrm{Concentration}\;(\mathrm{TPC})=\frac{\left(B\times D\right)}{V}=\;\mathrm{nmol}/\mu L$$

$$\left(B\right)\;represents\;the\;absorbance\;reading\;from\;the\;spectrophotometer.$$

$$\left(D\right)\;is\;the\;dilution\;factor,\;which\;is\;adjusted\;for\;any\;dilution\;performed\;during\;sample\;preparation.$$

$$\left(V\right)\;is\;the\;volume\;of\;the\;extract\;in\;microliters.$$

The final TPC concentration was expressed as pmol/µL.

### 2.6. TAC Assay

The total antioxidant capacity (TAC) was assessed using a TAC assay kit (STA-360, Cell Biolabs Inc., San Diego, CA, USA) and measured on a TECAN Infinite M200 PRO (Tecan US Inc, Morrisville, NC, USA) instrument with full-area, clear, 96-well flat-bottom plates (Greiner Bio-One, North America Inc, Summerfield, NC, USA). Duplicate aliquots of 25 µL of raw serum from each of the 72 samples, as well as 25 µL of the homogenized diet and PSP samples, were analyzed. Initial and final absorbances at 490 nm were recorded for both the samples and standards. Net absorbance values were used to create a uric acid standard curve, from which the uric acid equivalents (UAEs) of the samples were calculated. The μM copper-reducing equivalents (CREs), proportional to TAC, were then determined by multiplying the UAE concentrations by a factor of 2189 μM Cu++/μM uric acid.

### 2.7. Genomic DNA Extraction, Library Preparation, and 16S rRNA Gene Sequencing

Microbial DNA extraction and 16S rRNA library sequencing were conducted following a modified protocol described by Bledsoe et al. [39]. DNA was extracted from 72 mucosal samples (three individual fish samples from each of the 24 tanks) using the DNeasy 96 PowerSoil Pro kit (Qiagen Sciences Inc, Germantown, MD, USA) according to the manufacturer’s protocol. DNA concentrations were measured using a Nanodrop 2000 spectrophotometer (Thermo Fisher Scientific, Waltham, MA, USA). To ensure high purity (260/280 and 260/230 ratios of approximately 1.8), DNA samples were cleaned and concentrated using a gDNA Clean and Concentrate Kit (Zymo Research, Irvine, CA, USA).

Cleaned DNA samples were processed for library preparation using the Quick-16S™ Plus NGS Library Prep Kit (V3-V4) (Zymo Research) following the manufacturer’s protocol. 16S rRNA gene sequencing libraries were amplified using 341f and 806r 16S V3-V4 primers. The PCR conditions included a 10 min initial denaturation at 95 °C, followed by 42 cycles of 30 s denaturation at 95 °C, 30 s annealing at 55 °C, and 3 min elongation at 72 °C. 16S amplification was confirmed on a 2% agarose gel prior to equimolar pooling and 0.8× purification with Select-a-Size™ MagBeads (Zymo ResearchAfter purification, the pooled library was quantified using the NEBNext Library Quant Kit for Illumina (New England Biolabs, Ipswich, MA, USA), and DNA fragment analysis confirmed a final library size of approximately 600 bp. The ultra-pure pooled library was then processed for sequencing. The library was loaded on a MiSeq (Illumina, San Diego, CA, USA) system at a final concentration of 12 pM with 15% PhiX spike-in using the MiSeq Reagent Kit v3 (600 cycles) (Illumina).

After sequencing, raw reads were demultiplexed using Illumina’s Local Run Manager software (Version 3.0.0). To optimize quality control and ensure accurate downstream analysis, FIGARO [40] was employed to determine the ideal parameters for trimming the demultiplexed reads. These trimmed sequences were then imported into the DADA2 pipeline [41] within the R statistical environment [42]. DADA2 facilitated the correction of sequencing errors and the resolution of raw reads into high-resolution amplicon sequence variants (ASVs), representing unique biological sequences. Subsequently, chimeric sequences were removed, and taxonomic classification was assigned to the remaining amplicon sequence variants (ASVs) using the Silva nr99_v138.1 rRNA reference database [43]. ASVs were clustered based on sequence similarity using BLASTn [44], followed by post-clustering curation with LULU [45] to merge ASVs likely representing the same biological sequence, thereby mitigating potential overestimation of diversity due to sequencing artifacts.

Prior to statistical analysis, the dataset was refined by removing singletons, ASVs with a mean relative abundance of less than 1 × 10^−5^, and sequences assigned to the chloroplast order or the mitochondria family. Phyloseq [46] and vegan [47] packages were employed for data transformation and the calculation of ecological indices.

After quality control, 71 samples were retained for downstream analysis. Alpha diversity, measured by the Shannon index and observed ASVs, was compared across dietary pistachio shell powder (PSP) inclusion levels within the fishmeal and plant meal groups using a one-way ANOVA.

Rare taxa, defined as those with an abundance below 2 in at least 11% of the samples, were filtered out before beta diversity analysis. Cumulative sum scaling (CSS)-normalized count data were used for beta diversity assessment via principal coordinate analysis (PCoA) based on Bray–Curtis distances. Permutational Multivariate Analysis of Variance (PERMANOVA) with 9999 permutations (‘adonis2’ function) was used to identify significant differences in community composition between the fishmeal and plant meal groups, followed by pairwise ADONIS [48] comparisons with Benjamani–Hochberg correction. Additionally, beta dispersion was assessed (‘betadisper’ function) for group homogeneity, with ANOVA and pairwise permutation tests to detect dispersion differences.

The microbial community differences between dietary protein sources (fishmeal vs. plant meal) and increasing levels of pistachio shell powder (PSP) inclusion (0%, 0.5%, 1%, 2%) were assessed using an Analysis of Composition of Microbiomes (ANCOM-BC2) [49]. The analysis was performed on genus-level aggregated data, with taxa prevalence filtered to include only genera present in at least 20% of the samples. ANCOM-BC2 [49] was conducted with the default parameters, including Benjamani–Hochberg correction for multiple comparisons. Global tests for both overall differences in community composition and pairwise comparisons between PSP inclusion levels were performed.

### 2.8. RNA Extraction and Real-Time qPCR

The distal intestines of 72 fish samples were rinsed with PBS, snap-frozen in liquid nitrogen, and immediately transferred to a −80 °C freezer. Total RNA was extracted from the frozen distal intestinal samples using the Direct-zol RNA Miniprep kit (Zymo Research) following the manufacturer’s protocol. Samples were transferred to 2 mL lysis tubes containing 2 mm bashing beads (ZR BashingBeads, Zymo Research) and 1 mL of Trizol reagent and homogenized in a tissuelyzer (Mixer Mill 200, Retsch GmbH, Haan, Germany). The resulting lysate was then processed according to the manufacturer’s protocol. After extraction, the quality of the RNA was assessed using a Nanodrop 2000 spectrophotometer (Thermo Fisher Scientific). To ensure high purity (260/280 and 260/230 ratios of ≥1.8), RNA samples were purified using a one-step PCR inhibitor removal kit (Zymo Research).

Then, 1 μg of total RNA was taken from each sample, and DNase treatment followed by cDNA synthesis was performed using the iScript™ cDNA Synthesis Kit (BioRad, Hercules, CA, USA) according to the manufacturer’s protocol. Real-time quantitative PCR was performed on a CFX96 Real-Time System (BioRad) in reactions with a total volume of 10 μL. Two different supermixes were used: SsoAdvanced Universal Probes Supermix (BioRad), with 500 nmol primers and 300 nmol probes, and SsoAdvanced Universal SYBR Green Supermix (BioRad), with 500 nmol primers, following the manufacturer’s protocol. Each sample was run in duplicate for every fish. The PCR cycling conditions for all genes included an initial denaturation step at 95 °C for 3 min, followed by 40 cycles of denaturation at 95 °C for 5 s, and an annealing step at gene-specific temperatures (as detailed in Table 2), as per the method described by Bledsoe et al. [39]. Two reference genes, elongation factor 1α (Ef1-α) and β-actin, were used to calculate the geometric mean of their expression for normalization purposes. The relative expressions of genes involved in the oxidative stress response (NRF-2α, CAT, SOD, and GPX-1) and inflammatory response (TNF-α and S100) were determined using primers designed from rainbow trout sequences in the NCBI database (Table 2).

Primer PCR efficiency was determined using six serial dilutions of a standard pooled from representative samples across all experimental groups. On-plate primer efficiencies were used to correct Ct values for each individual run. Following quality control, where we failed to amplify one sample, 71 samples were retained for downstream analysis. Technical duplicates of samples had a coefficient of variation below 7%. Normalized data were analyzed using the relative quantification method established by Vandesompele et al. [50], as detailed below:

$$Relative\;gene\;expression=\frac{{\left(E_{GOI}\right)}^{\Delta Ct_{GOI}}}{\mathrm{GeoMean}\left[{\left(E_{REF}\right)}^{\Delta Ct_{REF}}\right]}$$

$$Converted\;primer\;efficiency=\left(\frac{\mathrm{Primer}\;\mathrm{efficiency}\;(\%)}{100}\right)+1$$

ΔCt=Calibrator Ct−Sample Ct

$$RQ=E^{\Delta Ct}$$

$$Relative\;gene\;expression=\frac{RQ_{GOI}}{\mathrm{GeoMean}\left[RQ_{REFs}\right]}$$

$$E=base\;of\;exponential\;amplification\;\left(i.e.,\;the\;efficiency\;of\;the\;reaction\right)$$

GOI=Gene of Interest

REF=reference genes

**Table 2:** RT-qPCR gene expression targets. Gene targets are listed with the corresponding NCBI accession used to design assays for the target, along with the primer and probe sequences used and the resulting efficiency (Primer Eff.) from each run.

Gene	Primer/Probe Sequence (5′–3′)	Primer Eff. (%)
Ef1-α NM_001124339.1	F: GTGAGTTTGAGGCTGGTATCT R: GCTCAGTAGAGTCCATCTTGTT P:/FAM/TGGGAGTGA/ZEN/AACAGCTCATTGTTGGA/3IABkFQ	Run 1: 95.95 Run 2: 105.27
β-actin NM_001124235.1	F: CTTCTCTCTCCACCTTCCAAC R: GGGATGGGTACAGTCTGTTTAG P:/FAM/CCTCCATCG/ZEN/TCCACCGTAAATGCT/3IABkFQ	Run 1: 104.63 Run 2: 104.11
TNF-α NM_001124357.1	F: CTGGGCTCTTCTTCGTTTACA R: GAGTCCGAATAGCGCCAAATA P:/FAM/AGGCTTCGT/ZEN/TTAGGGTCAAGTGCA/3IABkFQ	Run 1: 105.46 Run 2: 106.68
NRF-2α XM_036959401.1	F: GCAAGCTCATACTCTAGCTCTC R: CAGGGTTACTGTCCATCTCATC P:/FAM/TCCTTTGGT/ZEN/GGCTACAGCGATTCA/3IABkFQ	Run 1: 94.17 Run 2: 95.91
Ictacalcin S100I2 XM_036967731.1	F: GCTTGGAGAGATCATGGGGAAAA R: TCCACACTGCCATCTGCATTAG P:/FAM/ACACTGACC/ZEN/AGGCAAAGGTTGACA/3IABkFQ	Run 1: 102.58 Run 2: 107.31
SOD BT074393 [51]	F: CCACGGAGGACCCACTG R: CAGCTCCTGCAGTCACGTT P:/FAM/ACGTGCCGAACAGCAT/NFQ	Run 1: 99.67 Run 2: 106.18
CAT XM_021564310 [51]	F: GGACCTTACTGGCAACAACAC R: CGCTTCTGAGAGTGGATAAAGGAT P:/FAM/ACAGCATGGCGTCCCT/NFQ	Run 1: 95.23 Run 2: 99.44
GPX-1 NM_001124525.1 [52]	F: CGCCCACCCACTGTTTGT R: GCTCGTCGCTTGGGAATG	Run 1: 112.31 Run 2: 100.68

### 2.9. Proximate Analysis

The proximate composition of the practical diets (Table 1), PSP, and the whole-body content of the initial and final fish were analyzed following protocols adapted by Hong et al. [37]. Moisture levels were determined by drying feed and fish samples to a constant weight at 105 °C for 12 h. The dried samples were finely ground using a blender (magic bullet MBR-1101) and kept for subsequent crude protein and crude lipid composition analyses. Crude protein content, calculated as total nitrogen content (N) × 6.25, was analyzed using the combustion method with a nitrogen determinator (Elementar Rapid N Analyzer, Ronkonkoma, NY, USA). The crude lipid content was quantified using an ANKOM XT15 Extraction System (ANKOM Technology, Macedon, NY, USA). Samples were extracted with petroleum ether at 90 °C for 60 min. Following extraction, samples were dried at 102 °C for 30 min, cooled to room temperature in a desiccator, and weighed to determine the crude lipid content. Ash content was determined by incinerating samples at 600 °C for four hours in a muffle furnace. The caloric content of the samples was measured using an isoperibol bomb calorimeter (Parr 6300, Parr Instrument Company Inc., Moline, IL, USA). The fiber content of PSP was analyzed using the AOAC 2011.25 method, with slight modification [53].

### 2.10. Histology

Distal intestinal tissues were processed using protocols described by Hong et al. [37]. Samples were initially preserved in 10% neutral buffered formalin at 4 °C for 18 h before being rinsed in 2× PBS and transferred to 70% ethanol for long-term storage at 4 °C. Histological samples were processed at the Washington Animal Disease Diagnostic Lab (WADDL; Washington State University, Pullman, WA 99164, USA). Following standard histological procedures, the tissues underwent graded dehydration, xylene clearing, and paraffin embedding. Five-micrometer sections were obtained and stained with hematoxylin and eosin (H&E) for morphological assessment. Digitized slide images were subsequently analyzed using QuPath software (Version 0.5.0) [54] to quantify and evaluate intestinal morphology. Intestinal morphometric parameters, including villus length and width, were measured in seven replicate sections per fish. Following measurement, morphometric data from each fish were averaged within their respective tanks. For each dietary treatment group, four replicate measurements were randomly selected for subsequent histological analysis.

### 2.11. Data Wrangling and Statistical Analysis

In this study, a comprehensive suite of R (Version 4.4.1) packages was utilized to transform, wrangle, and analyze the data. High-resolution sample inference from amplicon data was achieved with DADA2 (Version 1.32.0) [41], while data manipulation and visualization were facilitated by the tidyverse (Version 2.0.0) [55] collection of packages. Phyloseq (Version 1.48.0) [46] was employed for comprehensive microbiome analysis, lulu (Version 0.1.0) [45] for resolving OTU clusters, and msa (Version 1.36.0) [56] and Biostrings (Version 2.72.1) [57] for multiple sequence alignment and string manipulation. Multipanel figures were assembled using patchwork (Version 1.2.0) [58] and cowplot (Version 1.1.3) [59].

For multiple comparison procedures, multcomp (Version 1.4.26) [60] and multcompView (Version 0.1.10) [61] were utilized to assess the statistical significance of observed differences. Phylogenetic analysis was conducted using ape (Version 5.8) [62], while forcats (Version 1.0.0) [63] streamlined categorical data manipulation. The vegan package (Version 2.6.6.1) [47] provided a suite of tools for ecological data analysis, and broom (Version 1.0.6) [64] was employed to convert statistical analysis objects into tidy data structures.

Differential abundance testing was performed using ANCOMBC (Version 2.6.0) [49], a specialized package for microbiome analysis. Heatmaps for visualizing differential abundance were generated using pheatmap (Version 1.0.12) [65]. Additionally, MetagenomeSeq [66] was utilized for cumulative sum scaling transformation prior to beta diversity analysis.

Univariate statistical tests were conducted separately for the fishmeal and plant-based groups using one-way ANOVA. This was followed by Welch’s *t*-tests to assess the impact of each treatment group (0.5%, 1%, and 2% PSP) relative to the control group (0% PSP) within each dietary category. One-way ANOVA was selected as the appropriate method for this study, given its focus on assessing the potential improvements PSP inclusion might offer in either a conventional fishmeal-based diet or an ultramodern plant meal-based diet independently, rather than investigating the interactive effects between PSP dosage and protein source. This approach was appropriate given that the diets were formulated to be isonitrogenous and isolipidic within each base formulation but varied slightly between formulations.

## 3. Results

### 3.1. Growth Performance and Whole-Body Proximate Analysis

At the conclusion of the feeding trial, the rainbow trout exhibited a substantial growth increase, approximately 1100%, with the mean initial weight of around 19 g increasing to a mean final weight of approximately 230 g. Detailed metrics on growth performance and nutrient utilization efficiency across the experimental diets are presented in Table 3 and Table 4, respectively. In plant meal-based diets, there were no significant differences in the growth and nutrient utilization efficiencies by PSP dose. However, for fishmeal-based diets, there were significant differences in AWG, SGR, and DGI, with fish fed a fishmeal diet at 1% PSP inclusion (FM3) having the highest AWG, SGR, and DGI while having the lowest FCR. Whole-body proximate analysis showed no significant differences in the moisture, protein, ash, or energy content among the dietary treatments for both FM and PM diets. Similarly, the protein efficiency ratio (PER), protein retention, and energy retention remained unaffected by PSP inclusion. The proximate analysis of the PSP revealed the ingredient to be rich in total fiber (86.9%), with most of the fiber content being insoluble (85.3%). Additionally, the PSP also contained small amounts of crude protein (3.8%) and crude lipid (2.5%).

### 3.2. Intestinal Gene Expression

**Figure 1:**
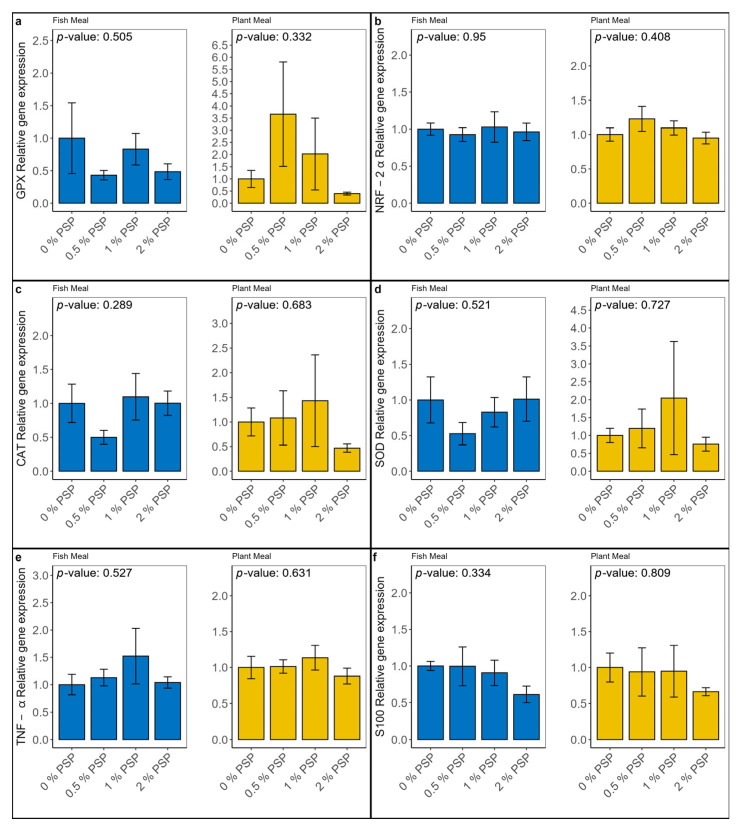
Relative mRNA expression of genes involved in oxidative stress—GPX, NRF-2α, CAT, and SOD (**a**–**d**, respectively)—and inflammatory response—TNF-α and S100 (**e**,**f**, respectively)—in the distal intestine of rainbow trout fed fishmeal or plant meal-based diets with varying levels of pistachio shell powder (PSP) inclusion (0%, 0.5%, 1%, 2%). *p*-values indicate the significance of differences between PSP inclusion levels within each diet type (one-way ANOVA). Y-axis represents relative expression.

Dietary inclusion of pistachio shell powder (PSP) did not significantly affect (*p* > 0.05) the relative mRNA expression of genes involved in oxidative stress (GPX-1, NRF-2α, CAT, SOD; Figure 1a–d) and the inflammatory response (TNF-α, S100; Figure 1e,f) in the distal intestine of rainbow trout. However, a trend towards the downregulation of genes involved in the inflammatory response was observed at the highest PSP inclusion level (2%) in the plant-based diet group.

### 3.3. TAC and TPC Analyses

**Figure 2:**
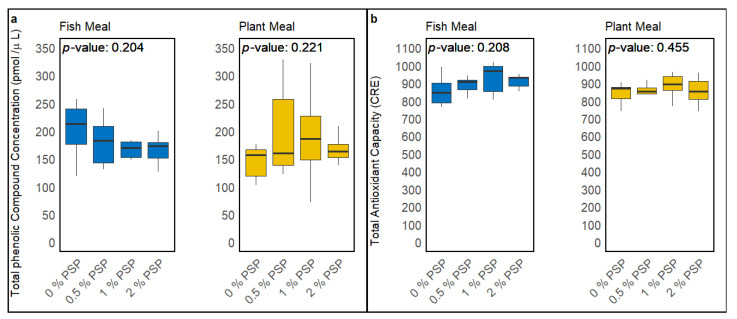
Effect of dietary pistachio shell powder (PSP) inclusion on total phenolic compound (TPC) concentration (**a**) and total antioxidant capacity (TAC) (**b**) in the serum of rainbow trout fed fishmeal or plant meal-based diets at different PSP inclusion levels (0%, 0.5%, 1%, 2%). *p*-values indicate the significance of differences between PSP inclusion levels within each diet type (one-way ANOVA). CRE—μM copper-reducing equivalent, which is proportional to TAC.

The dietary inclusion of pistachio shell powder (PSP) did not significantly alter the total phenolic compound concentration (TPC) or total antioxidant capacity (TAC) in the serum of rainbow trout fed either fishmeal-based or plant-based diets. In fish fed fishmeal diets, a slight decrease in the TPC and a slight increase in the TAC were observed with increasing PSP inclusion levels; however, these trends were not statistically significant (*p* = 0.204 for TPC, *p* = 0.208 for TAC; Figure 2a,b). Similarly, in the serum of fish fed plant-based diets, increasing PSP inclusion levels resulted in a minor increase in both the TPC and TAC, although these effects were also not statistically significant (*p* = 0.221 for TPC, *p* = 0.455 for TAC; Figure 2a,b).

**Figure 3:**
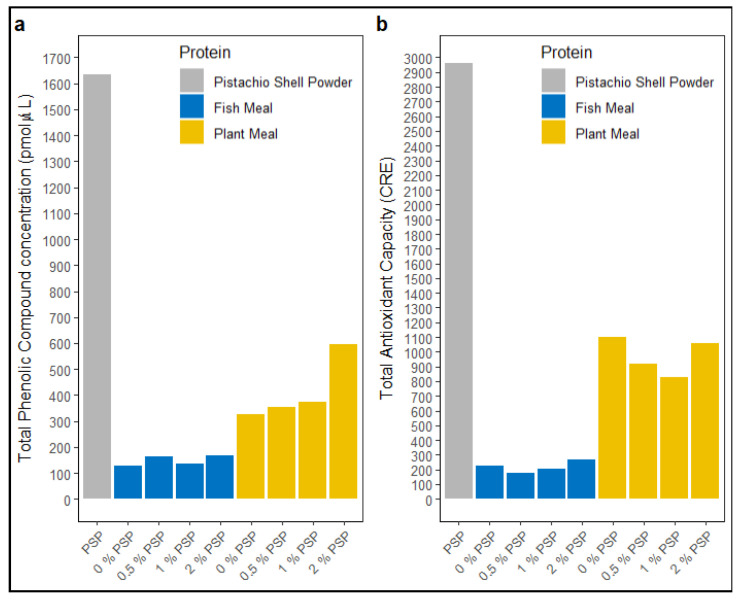
Total phenolic compound (TPC) concentration (**a**) and total antioxidant capacity (TAC) (**b**) in pistachio shell powder (PSP), fishmeal, and plant meal diets at different PSP inclusion levels (0%, 0.5%, 1%, 2%).

Dietary pistachio shell powder (PSP) showed a total phenolic compound concentration (TPC) of ~1635 pmol/μL (Figure 3a) and a total antioxidant capacity (TAC) of ~2964 CRE (Figure 3b). The inclusion of PSP in fishmeal diets did not affect TPC or TAC levels (Figure 3a,b). However, in plant meal-based diets, TPC levels increased with increasing PSP inclusion (Figure 3a).

### 3.4. Alpha Diversity

**Figure 4:**
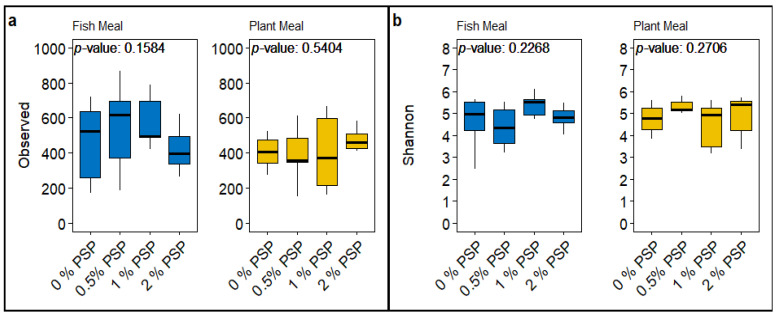
A comparative analysis of microbial alpha diversity indices across different dietary treatments containing either fishmeal or plant meal, each supplemented with varying levels of pistachio shell powder (PSP) inclusion (0%, 0.5%, 1%, 2%, and 4%). Panel (**a**) displays box plots representing the “Observed” species richness within the gut microbiomes of the sampled groups. Panel (**b**) illustrates the “Shannon” diversity index, a measure of both the richness and evenness of species present within the microbiomes.

The inclusion of pistachio shell powder (PSP) in both fishmeal- and plant meal-based diets did not significantly affect the alpha diversity of the gut microbiota in rainbow trout, as measured by the observed ASVs (amplicon sequence variants) and Shannon diversity index. In the fishmeal diets, there were no significant differences in either the observed ASVs (*p* = 0.1584; Figure 4a) or the Shannon diversity (*p* = 0.2268; Figure 4b) across varying levels of PSP inclusion (0%, 0.5%, 1%, 2%). This suggests that PSP did not substantially alter the richness or evenness of bacterial species in the gut of fish fed with fishmeal. Similarly, in the plant meal-based diets, the inclusion of PSP did not significantly influence the observed ASVs (*p* = 0.5404; Figure 4a) or Shannon diversity index (*p* = 0.2706; Figure 4b).

Overall, these results suggest that PSP inclusion, up to 2%, does not have a major impact on the alpha diversity of the gut microbiota in rainbow trout, regardless of whether the basal diet is fishmeal- or plant-based.

### 3.5. Beta Diversity

**Figure 5:**
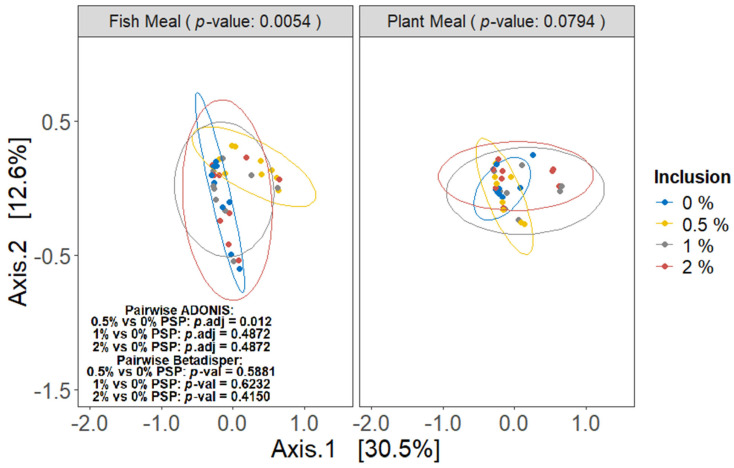
Beta diversity of gut microbiota as influenced by different dietary protein sources and levels of pistachio shell powder (PSP) inclusion, assessed using Bray–Curtis dissimilarity metrics with ADONIS test. The analysis is presented in two principal coordinate analysis (PCoA) plots, one for fishmeal (**left**) and one for plant meal (**right**). Individual-sample beta diversity is represented by points, colored according to the dietary PSP inclusion level (0%, 0.5%, 1%, or 2%) being received by that individual.

Pistachio shell powder (PSP) inclusion significantly altered the beta diversity of the gut microbiota in rainbow trout fed fishmeal diets (ADONIS, *p* = 0.0054; Figure 5), with the 0.5% inclusion level differing significantly from the control (pairwise ADONIS, *q* = 0.012; Figure 5). However, no significant differences were observed between the control group and other groups. Conversely, in fish fed plant-based meals, PSP inclusion did not significantly affect beta diversity (ADONIS, *p* = 0.0794; Figure 5), although the visual separation in the PCoA plot suggests potential subtle shifts in community composition. The dispersion of data points among the PSP inclusion levels was homogeneous in both diet types (betadisper, *p* > 0.05; Figure 5).

### 3.6. Differential Abundance

**Figure 6:**
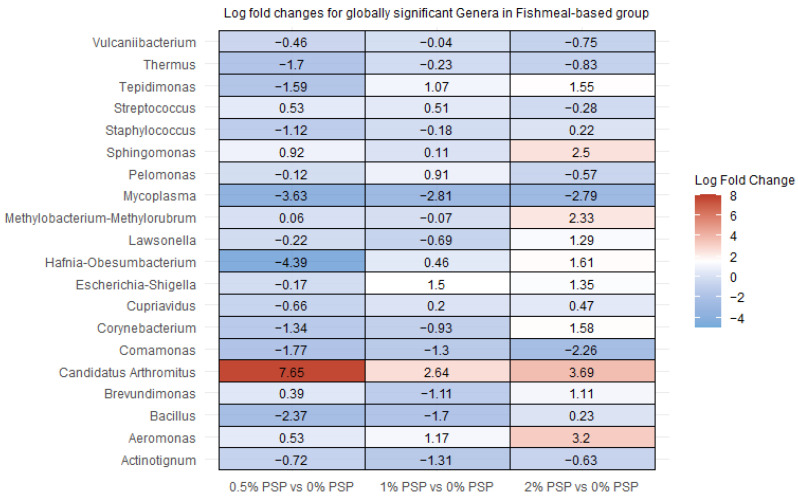
Differential abundance of bacterial genera in the distal intestines of fish fed fishmeal-based diets with varying levels of pistachio shell powder (PSP) inclusion.

**Figure 7:**
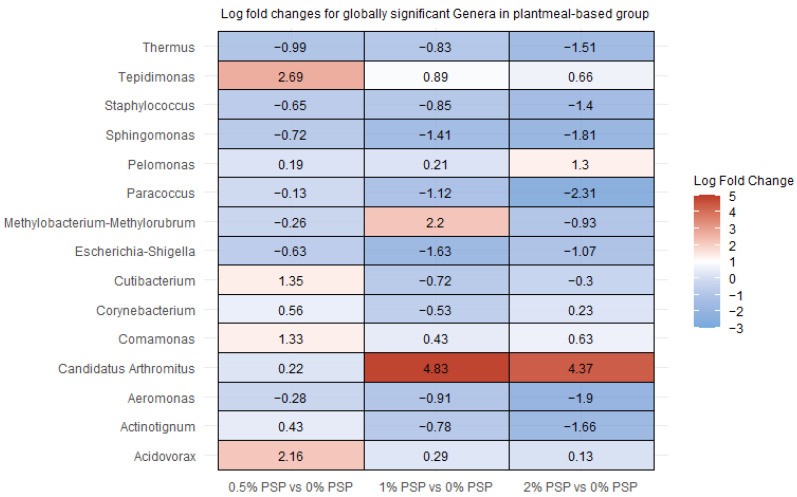
Differential abundance of bacterial genera in the distal intestines of fish fed plant meal-based diets with varying levels of pistachio shell powder (PSP) inclusion.

In rainbow trout fed a fishmeal-based diet, the inclusion of pistachio shell powder (PSP) induced significant alterations in the relative abundance of several gut bacterial genera. Specifically, *Candidatus arthromitus* showed a marked increase in abundance (Figure 6 and Figure 7) at all levels of PSP inclusion (0.5%, 1%, and 2%) compared to the control group (0% PSP). In contrast, *Mycoplasma* was significantly depleted with increasing PSP levels, while *Hafnia-Obesumbacterium* was significantly depleted at the 0.5% PSP inclusion level but enriched at the 1% and 2% inclusion levels (Figure 6). The magnitude and direction of these changes were dependent on PSP concentration. For instance, *Thermus* and *Comamonas* (Figure 6 and Figure 7) exhibited a consistent decrease at all PSP inclusion levels, whereas *Bacillus* (Figure 6) increased only at the highest PSP inclusion level (2%). Additionally, *Sphingomonas* and *Aeromonas* demonstrated a positive correlation with PSP inclusion, increasing in abundance as PSP levels increased.

In rainbow trout fed a plant-based diet, pistachio shell powder (PSP) inclusion resulted in significant alterations in the relative abundance of several gut bacterial genera. *Tepidimonas* and *Candidatus arthromitus* showed notable increases with PSP inclusion, particularly at 1% and 2% PSP, respectively (Figure 6 and Figure 7). In contrast, *Paracoccus* (Figure 7) displayed significant decreases at all PSP inclusion levels while *Sphingomonas* displayed significant decreases at all PSP inclusion levels in the PM group (Figure 7), and significant increases at all PSP inclusion in the FM group (Figure 6). Other genera, such as *Thermus*, *Staphylococcus*, and *Aeromonas*, showed mixed responses, with decreases at certain PSP concentrations and increases or no change at others.

### 3.7. Histology

**Figure 8:**
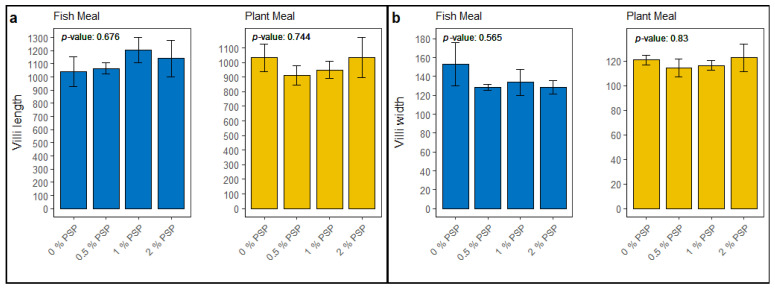
Morphometric analysis of intestinal villi in fish subjected to different dietary treatments with comparisons made across diets containing fishmeal and plant meal, supplemented with 0%, 0.5%, 1%, and 2% pistachio shell powder (PSP). Panel (**a**) depicts the villi length, and panel (**b**) illustrates the villi width.

The inclusion of pistachio shell powder (PSP) in the diet did not significantly affect the intestinal morphology of rainbow trout. In fish fed fishmeal diets, no significant differences (*p* > 0.05) were observed in villi length (Figure 8a) or villi width (Figure 8b) across varying levels of PSP inclusion (0%, 0.5%, 1%, and 2%). Similarly, in fish fed plant-based meals, PSP inclusion did not significantly influence villi length (Figure 8a) or villi width (Figure 8b).

## 4. Discussion

The pursuit of sustainable aquaculture has driven the exploration of alternative protein sources for fish diets. While these alternatives, primarily plant-based meals, offer economic benefits, they can also lead to adverse health effects in carnivorous fish species such as rainbow trout [67,68]. To address this, pistachio shell powder (PSP), a readily available by-product rich in antioxidants and fiber [14], was proposed as a potential functional feed ingredient in this study. Although some studies have evaluated the effects of other pistachio by-products on rainbow trout [69] and some herbivorous fish species [29,70,71], this study is the first to comprehensively evaluate the dose-dependent effects of PSP inclusion on growth performance, intestinal health, and gut microbiota modulation in rainbow trout fed either traditional fishmeal (FM) or plant meal-based (PM) diets. By examining these parameters across varying PSP concentrations within each dietary regimen, this research illustrates the effects of PSP supplementation in rainbow trout culture, contributing to the overall development of sustainable aquafeeds.

### 4.1. Growth Performance and Whole-Body Proximate Analysis

PSP supplementation at 1% inclusion significantly improved weight gain and growth rates in fish fed a fishmeal diet. This could be attributed to the presence of beneficial bioactive compounds in PSP, such as antioxidants and prebiotic fibers, potentially enhancing nutrient absorption and utilization. However, the lack of growth-promoting effects in fish fed a plant-based diet suggests that the protein source may modulate the response to PSP. The plant-based diets, rich in fiber, polyphenols, and antioxidants, as demonstrated by their high TPC and TAC content, may potentially mask or interact with the effects of PSP. Considering this, the inclusion of PSP likely provided no additional benefits in the plant meal-based diets as they were already well fortified with sufficient antioxidants, polyphenols, and fiber (Figure 3), rendering the supplementary effects of PSP redundant. The findings from this study align with some studies carried out in other animals. For instance, when Holstein calves were fed a diet supplemented with 6% pistachio by-product silage (PBPS), the average daily weight gain and FCR were significantly improved without affecting dry matter intake, suggesting that PBPS improved feed conversion efficiency [72]. In Kermanian male lambs, the inclusion of pistachio by-products (PBPs)—comprising soft hulls, twigs, leaves, hard shells, and green kernels—at a concentration of up to 20% had no significant effect on growth performance. However, when PBP levels exceeded 20%, a significant decline in growth performance metrics was observed [73]. In gestating sows, pistachio inclusion of up to 20% did not have negative effects on the health of the sows, and the gestating sows showed higher gross energy digestibility for PSP (60.9%) due to their extended digestive transient time compared to lactating sows with a GE ATTD of only 34.59% [74]. In Nile tilapia, an omnivorous fish species with a longer gut retention time, pistachio hull-derived polysaccharide (PHDP) inclusion at 0.5% and 1% was shown to significantly improve growth performance and nutrient utilization efficiency [30].

In the present study, PSP inclusion at 1% improved growth performance in fish fed the FM diets; however, no significant differences were observed in the whole-body proximate analysis in both the FM and PM groups. While the PM groups exhibited impressive growth rates, as evidenced by the average weight gain (Table 3), it is important to note that the FM groups demonstrated better overall nutrient utilization efficiency, as indicated by their lower FCR (Table 3). Additionally, it was observed that fish from the PM groups had a higher amount of visceral fat, which might have contributed to some of the weight gain observed. Although this study did not measure the viscerosomatic index (VSI), this might be an important metric to include during future investigations. No direct comparisons were made between the FM and PM groups because the goal of the experiment was not to assess the interactive effects of pistachio inclusion and protein source (PM vs. FM) on rainbow trout performance. Rather, the aim was to evaluate how PSP inclusion would perform across two distinct diet formulations based on different protein sources: (1) a conventional FM diet and (2) an advanced PM diet formulated with premium plant ingredients.

Although, similar to the trend seen in growth performance, the fish fed fishmeal diets containing 1% PSP inclusion had the best protein and energy retention efficiency ratios, albeit not statistically significant. The results from this study align with previous studies investigating the effects of antioxidant inclusion in rainbow trout diets, where the inclusion of antioxidants did not significantly affect the proximate composition [75,76] or protein efficiency ratios.

### 4.2. Intestinal Gene Expression and Histology

The PSP did not significantly alter the expression of oxidative stress or inflammatory genes in either the fishmeal-based diets or the plant meal-based diets. However, there was a trend toward the downregulation of inflammatory-related genes (TNF-α and S100) at 2% PSP inclusion in both the FM and PM groups and a trend toward the upregulation of antioxidant-related genes (SOD and CAT) at 1% PSP inclusion in the PM groups, suggesting potential anti-inflammatory or antioxidant effects. While there are limited studies highlighting the effects of pistachio by-products on immune-related genes, our findings show similar trends to the few related studies. For instance, in a study evaluating the effects of pistachio oil (PO) on a mouse macrophage cell line, PO at a maximum dosage of 5.5 mg/mL in the media was found to possess anti-inflammatory effects by reducing the expressions of lfit-2, TNF-α, IL-6 and IL-1β as the PO dose increased [77]. The same trend was observed in another study investigating the effects of pistachio hull polysaccharide (PHP) on inflammatory and immune responses in Nile tilapia, where the relative gene expressions of TNF-α, IL-1β, TLR2, Myd8 and NF-κB in the liver were downregulated as the PHP inclusion level increased [29].

All distal intestinal tissue had similar appearances, with no major differences observed across the groups. There were no significant differences observed in villi height and villi width across the plant-based and fishmeal-based groups. This could be attributed to the growing incorporation of plant proteins in commercial aquafeeds, potentially boosting the tolerance of commercial trout to plant-based diets, as observed in some selected lines of rainbow trout [7,78]. As a result, symptoms of soybean meal-induced enteritis (SBME) and other adverse effects linked to plant-based ingredients may not appear in the distal intestine until after prolonged exposure. Consequently, the 12-week trial utilized in this study may be insufficient to detect these changes, and future studies should focus on evaluating these effects in long-term feeding trials.

### 4.3. TAC and TPC Analyses

The investigation into the effects of pistachio shell powder (PSP) inclusion on the total phenolic compound concentration and antioxidant capacity in the serum of fish fed either a fishmeal-based diet or a plant meal-based diet showed no significant impact at the tested inclusion levels (0%, 0.5%, 1%, 2%). Interestingly, these findings contrast with those observed in a study involving dietary supplementation of polyphenols in common carp diets, where antioxidant activity significantly improved with increased inclusion of polyphenols [79]. In another study, supplementation of cello-oligosaccharides (COSs)—a dietary fiber source—improved antioxidant capacity in rainbow trout, as observed in serum [80]. Some other studies also demonstrate that the supplementation of trout diets with dietary antioxidants improves antioxidant capacity in serum [81,82,83].

These discrepancies may be attributed to several factors, including differences in the types of dietary supplements used, the bioavailability of the antioxidants and polyphenols, overall diet composition, the concentration of reactive oxygen species in the diet, and the species or strain of fish used in the studies. While PSP contains bioavailable antioxidants and polyphenols, it also has a high content of non-nutritive fiber, which may interfere with the absorption of these beneficial compounds in the gut [84], contrasting with studies where pure, extracted antioxidants were used, and the effects in the serum were more pronounced. Although there was an increase in dietary antioxidants and the phenolic compound content with higher PSP inclusion in both the FM- and PM-based diets, the presence of fiber and other non-active components in PSP could potentially reduce the efficacy of the antioxidants and polyphenols, thereby explaining the lack of significant improvements in the serum phenolic content and antioxidant capacity observed in this study.

### 4.4. Microbiome Analyses

Some studies evaluating the effects of antioxidants, dietary fiber, and other agricultural by-products on the gut microbiome of rainbow trout report increased microbial interactions, which lead to positive effects in overall fish health [80,83]. However, our finding that PSP did not significantly alter the alpha diversity of the gut microbiota aligns with the notion that carnivorous fish like rainbow trout may have limited microbial fermentation capabilities, which could reduce the impact of dietary fiber on gut microbial diversity [85]. Nevertheless, with the inclusion of PSP, the significant changes in beta diversity and the relative abundance of specific microbial taxa, such as *Candidatus arthromitus*, in FM diets indicate that PSP can influence the gut microbial community structure in a diet-specific manner.

The observed presence of *Candidatus arthromitus* within the gut microbiota is an interesting finding. *Candidatus arthromitus* is a segmented filamentous bacterium (SFB) known for its role in the immune system development of vertebrates [86]. In mammals, this bacterium adheres to the gut epithelium and can stimulate the host’s immune responses, including the production of IgA and the maturation of gut-associated lymphoid tissue (GALT) [87,88]. Although the modulation of *Candidatus arthromitus* in rainbow trout has not been extensively studied, the existing literature on its role in mammals could suggest similar functions in fish. Future shotgun metagenomic studies could provide deeper insights into the functional roles of this bacterium and other components of the microbiota in rainbow trout fed varying levels of PSP.

The observed reduction in the *Paracoccus* genus as the PSP levels in the PM diets decreased is another interesting discovery, as *Paracoccus*, a genus of soil microbes that has previously been isolated from carp [89], has been observed to be absent or present in very low abundance in the gut of healthy humans [90] but unusually abundant during diarrhea [91] and *Vibrio cholera* infection in humans [92]. In most studies involving humans or murine models, *Paracoccus* has been positively correlated with dysbiosis in the gut [93,94]. Given the limited studies on the function of *Paracoccus* in the rainbow trout gut, its potential function is not clear. However, our observations indicate a significant decrease in the differential abundance of *Paracoccus* as the inclusion levels of pistachio shell powder (PSP) increased up to 2% in fish fed plant-based diets. Since an increased abundance of *Paracoccus* has been associated with dysbiosis in other animal models, it can be hypothesized that the inclusion of PSP in the diet may promote gut normobiosis in rainbow trout, as evidenced by the reduced abundance of *Paracoccus*. Further research should be carried out to better understand the functions of key microbes in the gut of rainbow trout fed a PSP-based diet.

## 5. Conclusions

This study demonstrates that pistachio shell powder (PSP) inclusion in fish and plant meal diets up to 2% does not significantly affect the serum antioxidant capacity or phenolic compound concentration in rainbow trout. While PSP at 1% inclusion improved growth performance in fishmeal diets, no similar effect was observed in plant meal diets, suggesting a potential modulation by dietary protein source or at least overall dietary formulation. PSP inclusion did not significantly alter intestinal morphology or gene expression related to oxidative stress and inflammation, although trends toward anti-inflammatory and antioxidant effects were noted. Additionally, PSP inclusion influenced gut microbiota composition, particularly in fishmeal diets, highlighting its potential role in modulating gut health. These findings suggest that while PSP can be considered a safe dietary supplement, its benefits may be limited by its high fiber content and the overall diet composition, particularly in carnivorous fish species such as salmonids. Future research should aim to optimize PSP inclusion levels and investigate its long-term effects on additional compositional parameters, such as polyunsaturated fatty acids (PUFAs), essential amino acids, and lipophilic vitamins in the filet and whole body of rainbow trout. Furthermore, determining the optimal inclusion levels of PSP in various fish species, particularly herbivores, is essential to fully realize its potential in sustainable aquafeed production.

## Figures and Tables

**Table 1:** Dietary formulation and proximate composition of diets included in the twelve-week rainbow trout feeding trial.

Ingredient (%)	PM				FM			
	0%	0.5%	1%	2%	0%	0.5%	1%	2%
Pistachio shell powder	0	0.5	1	2	0	0.5	1	2
Soybean meal ^a^	25	25	25	25	--	--	--	--
Soy protein concentrate ^b^	23.43	23.43	23.43	23.43	--	--	--	--
Corn protein concentrate ^c^	10.23	10.23	10.23	10.23	--	--	--	--
Fishmeal ^d^	--	--	--	--	28.2	28.2	28.2	28.2
Poultry by-product meal ^e^	--	--	--	--	21.52	21.52	21.52	21.52
Blood meal ^f^	--	--	--	--	4.3	4.3	4.3	4.3
Wheat flour ^g^	13.3	12.8	12.3	11.3	27.56	27.06	26.56	25.56
Wheat gluten meal	2.24	2.24	2.24	2.24	--	--	--	--
Fish oil ^h^	17	17	17	17	14.4	14.4	14.4	14.4
Lysine HCl	1.85	1.85	1.85	1.85	1.12	1.12	1.12	1.12
Methionine	0.59	0.59	0.59	0.59	0.42	0.42	0.42	0.42
Threonine	0.32	0.32	0.32	0.32	0.58	0.58	0.58	0.58
Taurine ^i^	0.5	0.5	0.5	0.5	--	--	--	--
Dicalcium phosphate	2.75	2.75	2.75	2.75	--	--	--	--
Vitamin premix ^j^	1	1	1	1	1	1	1	1
Choline CL	0.6	0.6	0.6	0.6	0.6	0.6	0.6	0.6
Vitamin C ^k^	0.2	0.2	0.2	0.2	0.2	0.2	0.2	0.2
Trace min premix ^l^	0.1	0.1	0.1	0.1	0.1	0.1	0.1	0.1
Potassium chloride ^m^	0.56	0.56	0.56	0.56	--	--	--	--
Sodium Chloride	0.28	0.28	0.28	0.28	--	--	--	--
Magnesium oxide ^m^	0.05	0.05	0.05	0.05	--	--	--	--
Proximate composition								
Moisture (%)	6.36 ± 0.08	4.39 ± 0.12	3.77 ± 0.06	2.78 ± 0.03	3.45 ± 0.03	2.02 ± 0.05	1.22 ± 0.05	2.28 ± 0.07
Lipid—Dry weight (%)	15.37 ± 0.45	16.95 ± 0.24	16.14 ± 0.02	14.74 ± 0.08	19.61 ± 0.29	19.48 ± 0.01	18.78 ± 0.21	18.84 ± 0.05
Protein—Dry weight (%)	47.11 ± 0.13	45.43 ± 0.52	46.37 ± 0.48	47.53 ± 0.02	53.57 ± 0.14	54.17 ± 0.18	53.85 ± 0.20	54.29 ± 0.23
Ash—Dry weight (%)	6.54 ± 0.05	6.66 ± 0.21	6.81 ± 0.18	7.28 ± 0.10	3.21 ± 0.06	3.21 ± 0.06	3.37 ± 0.00	3.17 ± 0.02

^a^ Archer Daniels Midland Company, Chicago, IL, USA 472 g/kg protein; ^b^ Solae, Pro-Fine VF, St Louis, MO, USA 693 g/kg crude protein; ^c^ Cargill, Minneapolis, MN, USA, Empyreal 75, 756 g/kg crude protein; ^d^ Menhaden Special Select, Omega Proteins Corp, 610 g/kg crude protein; ^e^ IDF Inc., 832 g/kg protein; ^f^ Wilbur-Ellis, 892 g/kg crude protein; ^g^ Manildra Milling, 120 g/kg protein; ^h^ Omega Proteins Inc., Hammond, LA, USA, Virginia Prime menhaden oil; ^i^ NB Group Co. LTD., San Jose, CA, USA; ^j^ DSM Nutritional Products, Basel, Switzerland., ARS 702, per kg diet; vitamin A 9650 IU; vitamin D 6600 IU; vitamin E 132 IU; vitamin K3 1.1 gm: thiamin mononitrate 9.1 mg; riboflavin 9.6 mg; pyridoxine hydrochloride 13.7 mg; pantothenate DL-calcium 46.5 mg; cyanocobalamin 0.03 mg; nicotinic acid 21.8 mg; biotin 0.34 mg; folic acid 2.5 mg; inositol 600 mg.; ^k^ Stay-C, 35%, DSM Nutritional Products; ^l^ Sigma-Aldrich Company, St Louis, MO, USA, ARS 640, measured in mg/kg of diet; manganese 13; iodine 5; copper 9; zinc 40.; ^m^ Sigma-Aldrich Company. -- indicates an ingredient not present in that diet.

**Table 3:** Summary of the growth performance of rainbow trout fed either fishmeal (FM) or plant meal (PM)-based diets, supplemented with graded levels of pistachio shell powder (PSP: 0, 0.5, 1, 2%) for 12 weeks.

Diet	PSP Level	Initial Weight (g)	Average Weight Gain (g)	FCR	SGR	DGI	Survival	TGC
FM	0%	19.1 ± 0.26	186 ± 3.46	0.66 ± 0.02	3.01 ± 0.04	4.08 ± 0.05	100 ± 0.0	0.26 ± 0.01
FM	0.5%	19.07 ± 0.4	173.33 ± 1.53 *	0.71 ± 0.04	2.92 ± 0.03	3.92 ± 0.03	98.97 ± 1.79	0.26 ± 0.01
FM	1%	19.23 ± 0.32	190.33 ± 4.93 *	0.65 ± 0.02	3.02 ± 0.04	4.12 ± 0.06 *	95.87 ± 3.58	0.27 ± 0.01
FM	2%	19.17 ± 0.25	182.67 ± 3.21	0.72 ± 0.11	2.98 ± 0.02	4.04 ± 0.04	96.9 ± 0.0	0.26 ± 0.0
PM	0%	19.33 ± 0.23	236.33 ± 15.37	0.84 ± 0.1	3.27 ± 0.06	4.63 ± 0.15	97.93 ± 1.79	0.3 ± 0.01
PM	0.5%	19.3 ± 0.17	243.33 ± 12.66	0.8 ± 0.06	3.3 ± 0.06	4.71 ± 0.13	100 ± 0.0	0.31 ± 0.01
PM	1%	18.93 ± 0.25	240 ± 21.66	0.86 ± 0.03	3.31 ± 0.09	4.69 ± 0.21	98.97 ± 1.79	0.3 ± 0.02
PM	2%	19.1 ± 0.2	238 ± 17.35	0.83 ± 0.07	3.29 ± 0.08	4.66 ± 0.18	100 ± 0.0	0.3 ± 0.01
One-way ANOVA	FM: PSP level	0.918	*0.002*	0.434	*0.022*	*0.003*	0.119	0.163
	PM: PSP level	0.161	0.962	0.759	0.893	0.956	0.219	0.908

Data for each experimental group are reported as mean ± standard deviation from triplicate tanks. Results were subjected to a one-way ANOVA to specifically evaluate PSP inclusion within each diet type. FCR—feed conversion ratio; SGR—specific growth rate; DGI—daily growth index; TGC—thermal growth coefficient. Bold asterisks (*) represent significant differences from Welch’s *t*-test conducted between pistachio shell powder (PSP) inclusion levels (0.5, 1, 2%) and the control (0%), within fishmeal (FM) or plant meal (PM) diets.

**Table 4:** Summary of the whole-body proximate analysis of rainbow trout fed either fishmeal (FM)- or plant meal (PM)-based diets, supplemented with graded levels of pistachio shell powder (PSP: 0, 0.5, 1, 2%) for 12 weeks.

Diet	PSP Level	Whole Body Moisture	Whole Body Protein	Whole Body Ash	Whole Body Energy (cal)	Protein Efficiency	Protein Retention (%)	Energy Retention (%)
FM	0%	67.47 ± 0.59	15.6 ± 0.72	1.47 ± 0.07	2300.7 ± 34.26	3.09 ± 0.06	48.73 ± 2.9	65 ± 1.03
FM	0.5%	68 ± 0.36	15.97 ± 0.63	1.68 ± 0.13	2230.33 ± 40.21	2.86 ± 0.18	46.18 ± 1.14	58.87 ± 4.87
FM	1%	66.97 ± 0.54	15.72 ± 0.67	1.58 ± 0.11	2265.87 ± 111.28	3.2 ± 0.09	50.79 ± 1.53	65.39 ± 5.2
FM	2%	67.25 ± 0.74	15.75 ± 0.16	1.59 ± 0.11	2301.22 ± 127.79	2.85 ± 0.4	45.42 ± 6.43	59.78 ± 7.22
PM	0%	68.25 ± 0.63	16.19 ± 0.38	1.91 ± 0.03	2164.52 ± 74.12	2.87 ± 0.33	47.01 ± 6.22	57.06 ± 8.45
PM	0.5%	68.05 ± 0.51	16.68 ± 0.26	1.97 ± 0.09	2181.62 ± 30.68	3.04 ± 0.24	51.48 ± 4.77	56.43 ± 4.57
PM	1%	68.08 ± 0.81	16.68 ± 0.2	1.85 ± 0.31	2174 ± 63.31	2.75 ± 0.09	46.43 ± 1.69	51.94 ± 3.28
PM	2%	68.81 ± 1.36	16.76 ± 0.32	2.12 ± 0.21	2091.8 ± 91.13	2.79 ± 0.27	47.37 ± 5.16	50.5 ± 4.77
One-way ANOVA	FM: PSP level	0.239	0.888	0.200	0.734	0.235	0.323	0.327
	PM: PSP level	0.711	0.159	0.406	0.405	0.504	0.578	0.437

Data for each experimental group are reported as mean ± standard deviation from triplicate tanks. Results were subjected to a one-way ANOVA to specifically evaluate PSP inclusion within each diet type.

## Data Availability

The demultiplexed sample-specific 16S rRNA V3V4 gene amplicon data presented in the study are openly available in NCBI in BioProject PRJNA1146053, and data are available in the Sequence Read Archive at: https://www.ncbi.nlm.nih.gov/bioproject/PRJNA1146053 (accessed on 16 July 2024). Data analysis codes used in this study are available at: https://github.com/LordBanik/Pistachio-shell-powder-in-trout-diets (accessed on 16 July 2024).

## Abbreviations

**Abbreviation**	**Description**
PSP	Pistachio Shell Powder
FM	Fishmeal
PM	Plant Meal
TAC	Total Antioxidant Capacity
TPC	Total Phenolic Compounds
Ef1-α	Elongation factor 1α
β-actin	Beta actin
NRF-2α	Nuclear Factor erythroid 2-related factor 2a
CAT	Catalase
SOD	Superoxide dismutase
GPX-1	Glutathione peroxidase 1
TNF-α	Tumor Necrosis Factor-alpha
S100	Ictacalcin S10012
AWG	Average Weight Gain
ROS	Reactive Oxygen Species
PER	Protein Efficiency Ratio
SGR	Specific Growth Rate
SBME	Soybean Meal-Induced Enteritis
FCR	Feed Conversion Ratio
IACUC	Institutional Animal Care and Use Committee
DO	Dissolved Oxygen
PCR	Polymerase Chain Reaction
qPCR	Quantitative PCR
NGS	Next-Generation Sequencing
RQ	Relative Quantification
ASV	Amplicon Sequence Variant
DNA	Deoxyribonucleic Acid
RNA	Ribonucleic Acid
cDNA	Complementary DNA
PBS	Phosphate-Buffered Saline
ANOVA	Analysis of Variance
PERMANOVA	Permutational Multivariate Analysis of Variance
PCoA	Principal Coordinate Analysis
CSS	Cumulative Sum Scaling
ANCOM-BC:	Analysis of Composition of Microbiomes with Bias Correction
H&E	Hematoxylin and Eosin
